# Bilateral breast treatment in a single arc using RapidArc Dynamic: A planning comparison with conventional volumetric modulated arc therapy

**DOI:** 10.1002/acm2.70380

**Published:** 2025-11-21

**Authors:** Taoran Li, Luca Cozzi, Lesley Rosa, Ryan Clark, Priyanka Agarwal, Yin Gao, Yun Yang, Boon‐Keng Kevin Teo, Anthony Magliari, Himank Kalra, Francisco Reynoso, Patrick Kupelian, Sushil Beriwal

**Affiliations:** ^1^ Department of Radiation Oncology University of Pennsylvania Philadelphia Pennsylvania USA; ^2^ Varian Medical Systems Palo Alto California USA; ^3^ Humanitas Cancer Center and Research Hospital Rozzano Italy; ^4^ Washington University School of Medicine in St. Louis, Department of Radiation Oncology St. Louis Missouri USA; ^5^ Department of Radiation Oncology University of California Los Angeles California USA; ^6^ Allegheny Health Network Pittsburgh Pennsylvania USA

**Keywords:** delivery efficiency, RapidArc Dynamic (RAD), synchronous bilateral breast cancer (SBBC), treatment planning, volumetric modulated arc therapy (VMAT)

## Abstract

**Purpose:**

Synchronous bilateral breast cancer (SBBC) presents significant radiotherapy planning challenges due to complex target volumes near critical organs. This study evaluated RapidArc Dynamic (RAD), a novel technique integrating dynamic arcs with static angle modulated ports (STAMPs) via simultaneous GPU‐accelerated optimization, against conventional volumetric modulated arc therapy (VMAT) for SBBC treatment planning and delivery efficiency.

**Methods:**

Ten retrospective SBBC cases involving comprehensive nodal irradiation were planned using both multi‐arc VMAT (six arcs) and RAD (one arc plus six STAMPs) in Eclipse (v18.1) with AcurosXB. A prescription dose of 40.05 Gy in 15 fractions was normalized to PTV D95% = 95%. Paired Wilcoxon signed‐rank tests compared planning target volume (PTV) coverage (V95%, V105%) and organ‐at‐risk (OAR) doses, including lungs (V5Gy, V10Gy, V20Gy), heart (D_mean_, V15Gy), and esophagus (D_mean_, D0.03 cm^3^). Planning time, monitor units (MU), and measured delivery times were also assessed.

**Results:**

RAD plans achieved clinical goals more frequently than VMAT plans (90% vs. 70% of endpoints). PTV V95% coverage was comparable between the two techniques. However, RAD significantly improved breast dose homogeneity (lower median PTVp V105%; *p* < 0.05) and OAR sparing. Notably, RAD reduced median lung V5Gy by 9.7% (*p* < 0.001) and esophageal D0.03 cm^3^ by 5.4 Gy (*p* = 0.002). RAD significantly reduced planning time (avg 16 vs. 39 min), mean MU (1654 vs. 2268; ∼30% reduction), and mean measured delivery time (2.2 vs. 5.8 min; ∼62% reduction).

**Conclusion:**

For SBBC radiotherapy, RAD demonstrated significant dosimetric improvements over conventional VMAT, particularly reducing low‐dose lung and high‐dose esophageal irradiation while enhancing target homogeneity. The substantial gains in planning and delivery efficiency further establish RAD as a highly advantageous technique for managing these complex cases.

## 1 INTRODUCTION

Synchronous bilateral breast cancer (SBBC), occurring in approximately 2%–5% of breast cancer cases,^1^ poses significant radiation therapy challenges due to large, complex target volumes adjacent to critical organs at risk (OARs) like the heart, lungs, and esophagus.^2^, ^3^ Traditional tangential fields often result in dose inhomogeneity and inadequate OAR sparing.^4^ Advanced techniques like intensity‐modulated radiation therapy (IMRT) and volumetric‐modulated arc therapy (VMAT) improve dose conformity.^2^, ^5^ However, standard IMRT can be time‐consuming, while single‐arc VMAT may compromise target homogeneity or OAR sparing in complex cases, potentially requiring multiple arcs which increases delivery time and low‐dose spillage.^6^, ^7^

To balance plan quality and efficiency, “hybrid” techniques combining static fields (like 3D‐CRT or IMRT) with dynamic arcs (VMAT) have been explored.^4^, ^8^, ^9^ Systematic reviews suggest these hybrid approaches (H‐IMRT, H‐VMAT) can offer dosimetric advantages over conventional techniques in whole breast radiotherapy (WBRT), often by leveraging the strengths of both components.^8^, ^10^ For instance, H‐VMAT combining tangential beams with VMAT arcs has shown promise in reducing OAR doses while maintaining target coverage.^10^, ^11^ Strategies like FusionArc have formalized methods for selecting optimal angles for IMRT modulation within a VMAT framework.^9^ However, the optimal weighting and beam arrangement for hybrid plans remain areas of investigation^8^, ^9^ and many hybrid approaches involve sequential optimization, potentially limiting efficiency and benefit.^4^

Further refinements in rotational therapy have explored optimizing VMAT with dynamic collimator rotation (DC‐VMAT) during arc delivery.^12^, ^13^ Studies have shown that dynamically adjusting the collimator angle can enhance modulation capabilities, potentially improve dose conformity by aligning with target or OAR orientation, and achieve superior dosimetry compared to static collimator VMAT, even potentially mitigating disadvantages of larger MLC leaf sizes.^12^ However, implementing optimized DC‐VMAT has faced challenges due to the complexity of integrating collimator angle selection into the optimization process and ensuring mechanical deliverability within linac constraints. Early approaches often relied on geometric alignment rather than fully dose‐based optimization.^12^, ^13^, ^14^

RapidArc Dynamic (RAD, Varian Medical Systems, Palo Alto, California, USA) was introduced recently as an optional feature on TrueBeam version 4.1 and Eclipse version 18.1, which is a novel and automated approach integrating benefits from several of these approaches. It combines dynamic arc delivery with STAMPs (static angle modulated ports) using a single, simultaneous, GPU‐accelerated optimization algorithm.^15^ This integrated optimization theoretically overcomes limitations of sequential hybrid planning. RAD also incorporates benefits for dynamic collimator rotation^12^ and automatic skin flash generation^15^ aiming to produce highly conformal, homogeneous, and robust dose distributions while significantly reducing planning and delivery times compared to conventional VMAT or other hybrid techniques. Preliminary data suggest RAD offers substantial OAR sparing and efficiency gains for left breast, spine, and brain lesions.^16^, ^17^, ^18^, ^19^, ^20^ Given the complexities of SBBC, this study aimed to evaluate RAD's performance against conventional multi‐arc VMAT, hypothesizing improved dosimetry and efficiency based on its integrated optimization approach.

## 2 MATERIALS AND METHODS

### 2.1 Patient selection and simulation

This retrospective study included 10 patients diagnosed with SBBC who received VMAT‐based radiation therapy. Eligibility required treatment encompassing both breasts and chest walls, including delineated post‐lumpectomy tumor beds, and comprehensive regional lymphatics (axillary, supraclavicular, and upper three internal mammary nodes). All patients underwent CT simulation (2.5 mm slice thickness) in a supine position on a commercial breast board with both arms abducted and elevated above the head to ensure reproducible positioning.

### 2.2 Target volume and organ‐at‐risk delineation

Clinical target volumes (CTVs), including bilateral breast/chest wall, and regional nodal stations, were first automatically delineated using a commercially available auto‐segmentation software AI‐Rad Companion (Siemens Healthineers) and then reviewed by an experienced radiation oncologist following institutional guidelines. Tumor beds were contoured referencing surgical clips or surgical changes. Planning target volumes (PTVs) were generated by applying institutional standard margins to the CTVs to account for setup uncertainties and internal motion. Key OARs—including total lungs, heart, esophagus (from cricoid to tracheal bifurcation), spinal cord, LAD (when visible), and body contour—were also delineated using AI‐Rad Companion and then reviewed by the same radiation oncologist.

### 2.3 Treatment planning techniques

**FIGURE 1:**
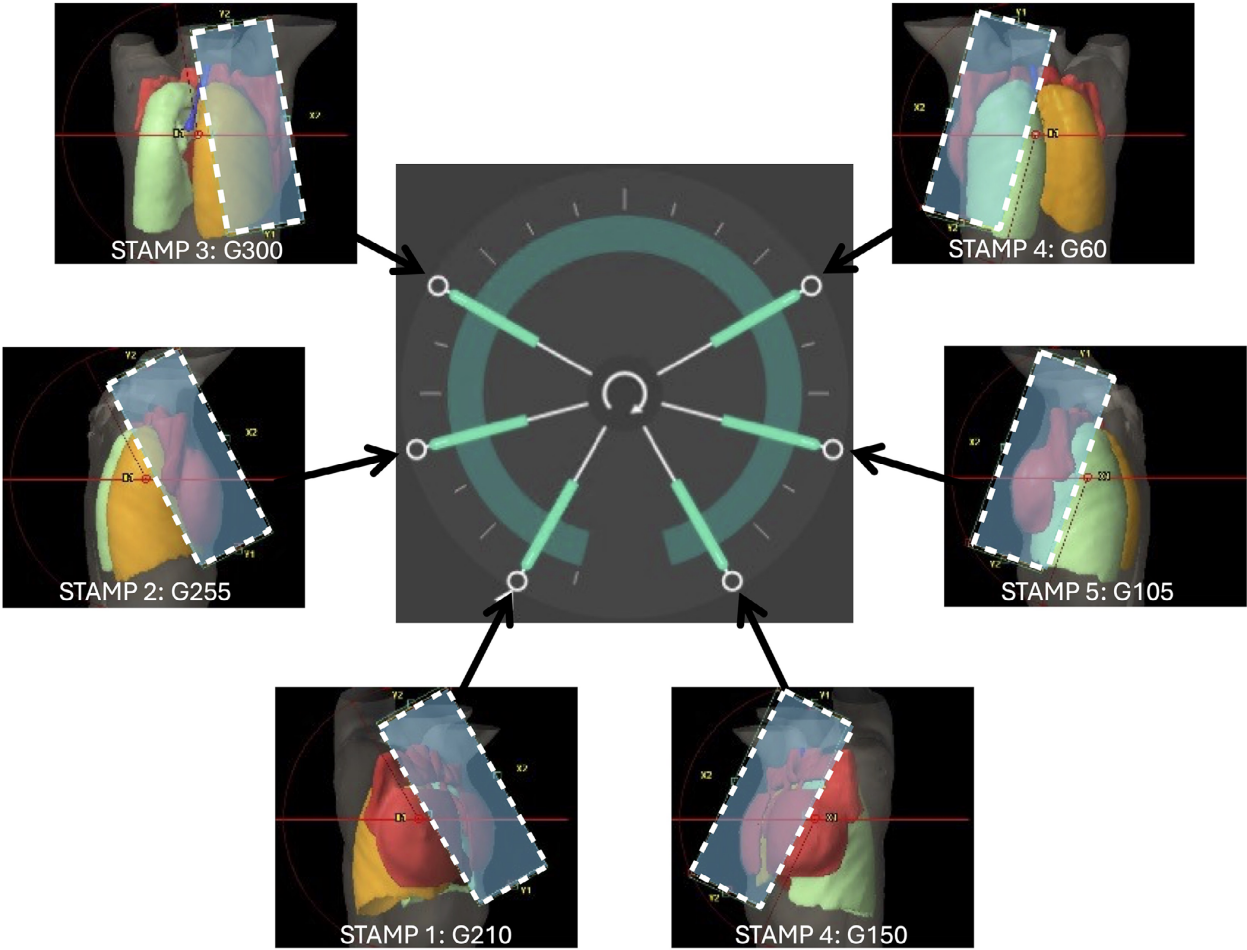
Beam arrangement template used for RapidArc Dynamic plans for all patients. Six STAMPs were shown with gantry angles and BEV of a representative patient. Blue rectangle with white dashed outline indicates collimator rotation and optimization aperture limit. Collimator dynamically rotates during arc portion by interpolating between static angles’ settings.

For each patient, two distinct treatment plans were generated by the same treatment planner using the Eclipse Treatment Planning System (Varian Medical Systems, Palo Alto, California, USA), version 18.1:

1. Conventional VMAT (VMAT): Plans utilized five to six coplanar partial arcs, with each collimator angle selected to maximize PTV coverage and allow for optimal OAR sparing. Arc entrance angles were tailored to encompass the extensive target volumes while avoiding contralateral beam entry where possible. Skin flash is incorporated using the widely accepted virtual bolus technique.^21^
2. RapidArc Dynamic (RAD): Plans consisted of a single, near full coplanar arc with six STAMPs (static angle modulated ports). The specific angles for the STAMPs were selected using a template and then fine‐tuned based on target geometry and OAR proximity. Dynamic collimator rotation and automatic skin flash features were also utilized in this specific comparison. Automated skin flash parameters were set at 7 mm expansion from skin with ‐350 HU value, as suggested by Hubley et al.^17^ Figure 1 shows the beam arrangement template used for all patients. Minor adjustments of STAMP angle and collimator angles were made based individual patients PTV and chest wall shape. But overall beam arrangement all followed this template.

Both planning techniques employed the photon optimizer (PO) and the AcurosXB (AXB) dose calculation algorithm (version 18.1) with a calculation grid size of 2.5 mm. A key feature of the RAD planning process is the use of a fully GPU‐accelerated optimizer that simultaneously optimizes apertures of the arc portion and the STAMPs, ensuring integrated and efficient dose delivery. All plans were prescribed a dose of 40.05 Gy in 15 fractions and normalized such that 95% of the prescribed dose covered 95% of the combined PTV (PTV_TOTAL_, encompassing all target volumes). Standard institutional OAR dose constraints were applied during optimization for both techniques (examples listed in Results table).

### 2.4 Dosimetric and efficiency evaluation

Treatment plans were evaluated using dose‐volume histograms (DVHs). Key dosimetric parameters were extracted for target volumes and OARs.

- Target coverage & homogeneity: PTV_TOTAL_ Homogeneity Index [HI = (D2%—D98%)/D50%], Conformity Index (CI), V95% (volume receiving ≥ 95% of prescription dose). PTVp (left and right primary tumor bed PTVs) V105% (volume of breast receiving ≥ 105% of prescription dose).
- OAR sparing: Lungs (V5Gy, V10Gy, V20Gy—volume receiving ≥ 5, 10, 20 Gy), Heart (Dmean, V15Gy, D5%), Esophagus (Dmean, D0.03 cm^3^—dose to 0.03 cc), LAD (D0.03 cm^3^), Spinal Cord (D0.03 cm^3^), Body (D0.03 cm^3^, V100%—volume receiving ≥ 100% of prescription dose in cc).

Planning efficiency was assessed by recording the active planning time (approximated as the time from the start of optimization to the completion of final dose calculation). Delivery efficiency was evaluated by comparing the total Monitor Units (MU) per plan and the actual plan delivery time on a TrueBeam 4.1 linac for three representative cases with typical plan complexity.

### 2.5 Delivery accuracy

Delivery of all RAD plans was performed on a TrueBeam 4.1 linac and analyzed using clinical standard Portal Dosimetry with gamma analysis (2 %/2 mm and 3 %/3 mm, global maximum‐dose normalization, 90% pass rate). To further quantify accuracy of various mechanical components, trajectory‐log files were analyzed of gantry, collimator, and MLC positions.

### 2.6 Statistical analysis

Dosimetric parameters, planning time, and delivery metrics (MU, delivery time) for the VMAT and RAD plans were compared using a paired, non‐parametric statistical test. Specifically, the Wilcoxon signed‐rank test was employed due to the paired nature of the data and the sample size (*N* = 10). A *p* value < 0.05 was considered statistically significant. Data analysis was performed using standard statistical software. Results for dosimetric parameters are primarily reported as median values with ranges, and differences are reported as median paired differences (VMAT—RAD) with ranges. Planning and delivery efficiency metrics are reported as mean ± standard deviation where appropriate, based on the available data points.

## 3 RESULTS

**FIGURE 2:**
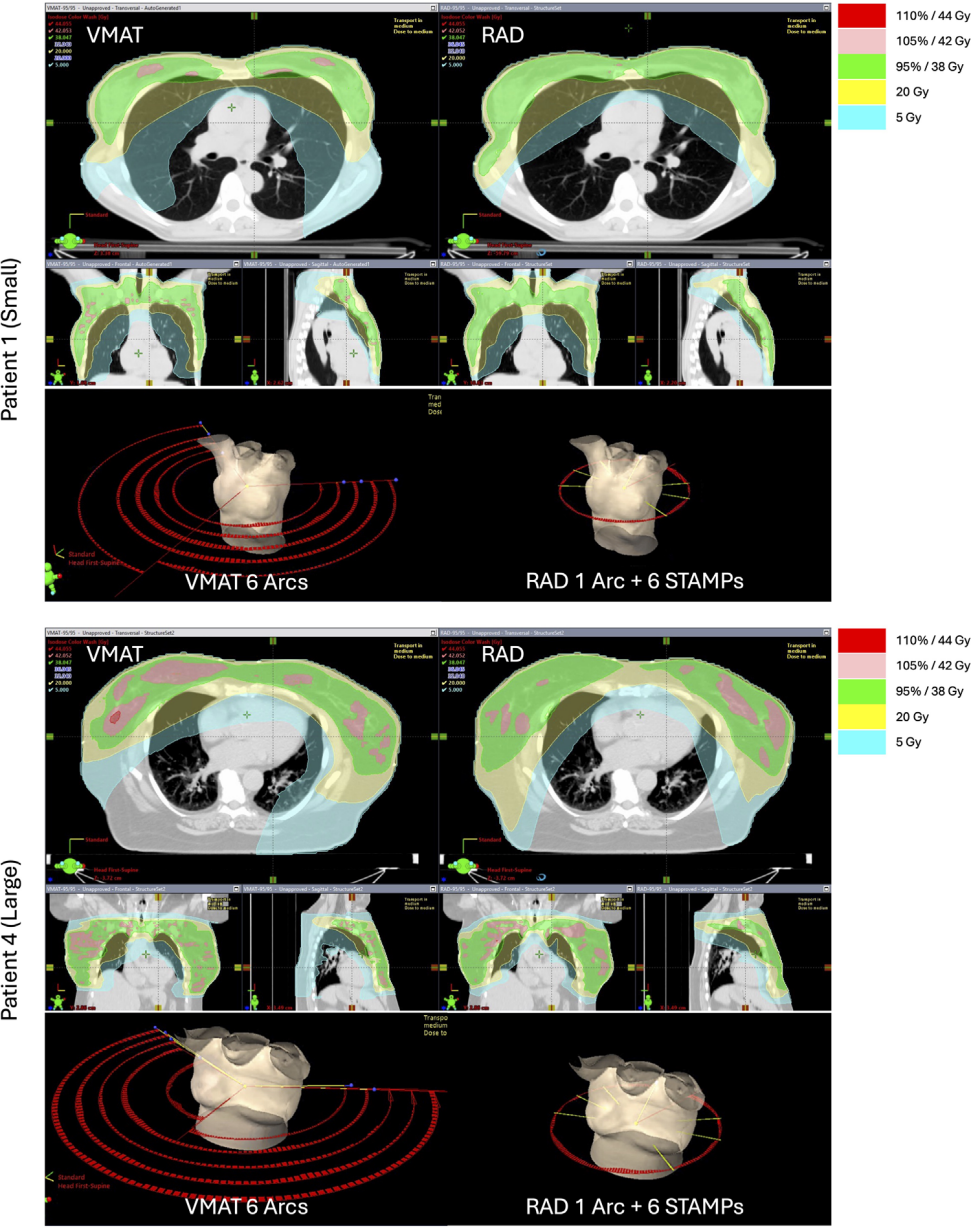
Representative cases showing small (upper) and large (lower) body habitus examples. Isodose color bands and beam/arc arrangements are shown for both VMAT (left) and RAD (right) plans.

**FIGURE 3:**
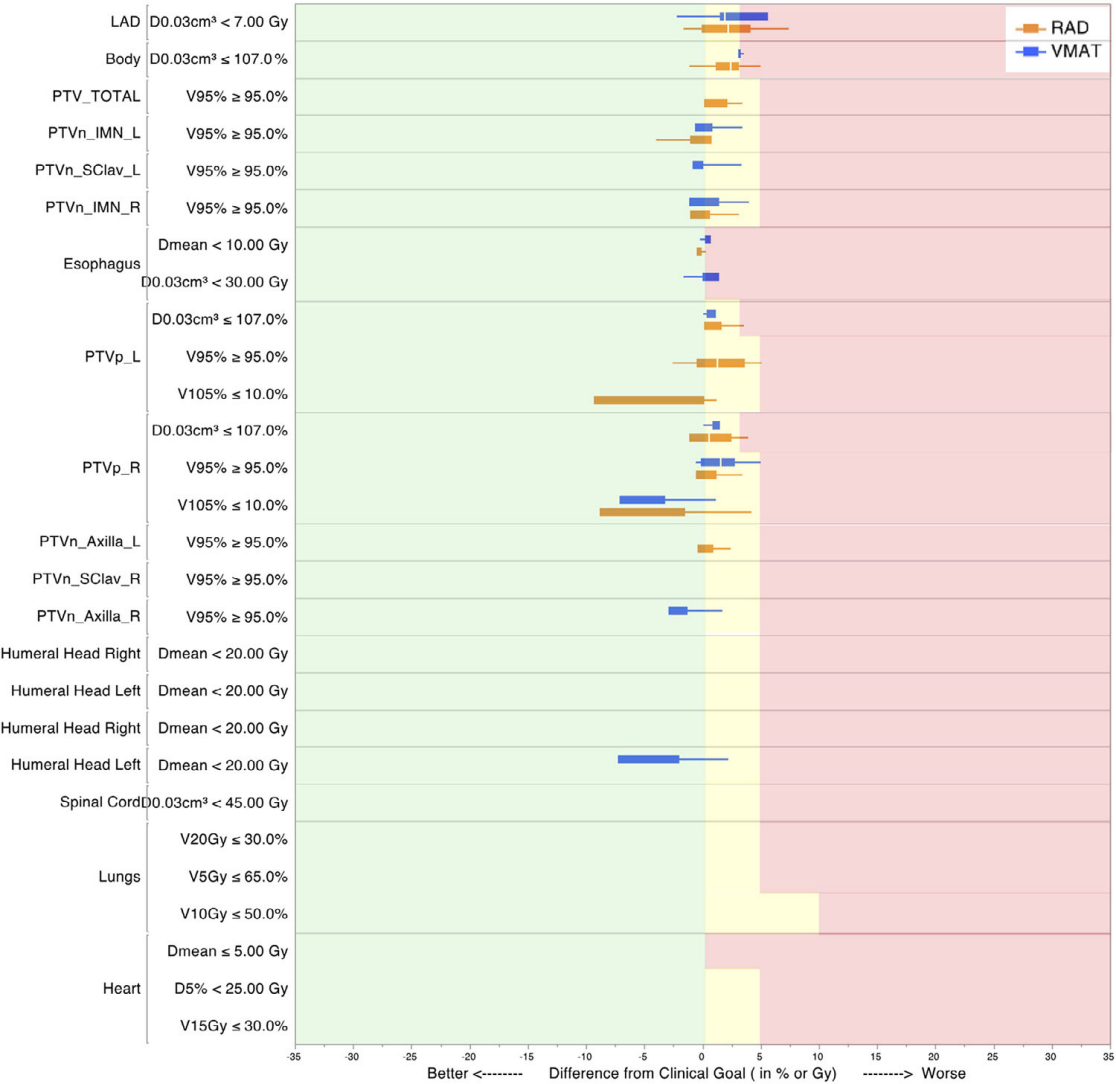
Comparison of key dosimetric endpoints achieved by VMAT (blue color) and RAD (orange color) against clinical goals for different DVH endpoints. Green means at or better than goals, yellow means within variation acceptable range, and red means not met.

RAD plans demonstrated superior overall quality compared to VMAT plans, meeting or exceeding clinical goals for 90% of evaluated DVH endpoints, compared to 70% for VMAT. Figure 2 shows two representative patient cases with relatively small and large body habitus. Notice the smaller 5 Gy band in RAD plans compared to VMAT plans, as well as smaller V105% (pink) regions in RAD plans. Figure 3 summarizes all the dosimetric parameters evaluated, showing RAD (orange bars) consistently achieving goals across most parameters, particularly for OARs like esophagus and lungs, while VMAT (blue bars) showed more frequent deviations, especially for certain OAR and target homogeneity metrics.

**FIGURE 4:**
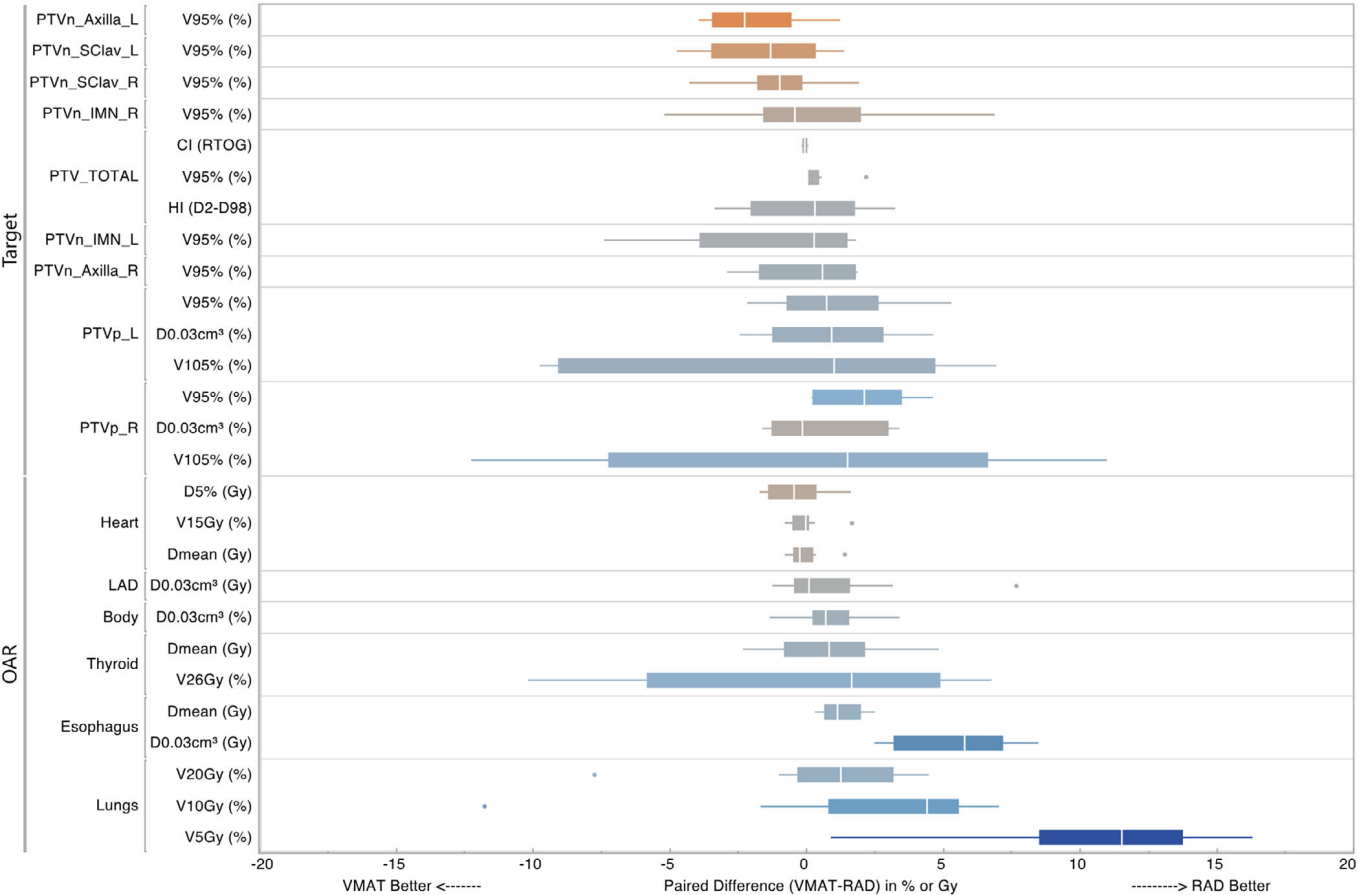
Paired comparison between original VMAT plan and RAD plan. Each same in the boxplot is calculated by subtracting the DVH endpoint of RAD plan from the corresponding VMAT plan from the same subject. This way any positive data suggesting RAD is better, while negative suggests RAD is worse than VMAT for that particular DVH endpoint.

While both techniques achieved clinically acceptable and comparable target coverage (PTV_TOTAL_ V95%), RAD plans offered significant dosimetric advantages for key OARs and target homogeneity. The most substantial improvements were observed for esophageal sparing and reduction of low‐dose lung irradiation. RAD significantly lowered the near‐maximum esophageal dose (D0.03 cm^3^ reduced by 5.4 Gy, *p* = 0.002) and the lung V5Gy (median reduced by 9.7%, *p* < 0.001). The paired difference plot in Figure 4 illustrates the magnitude and consistency of these benefits, with the majority of data points for Esophagus D0.03 cm^3^ and Lungs V5Gy showing positive differences (favoring RAD). In contrast, differences for lung V10Gy and V20Gy were smaller and not statistically significant (Table 1).

**TABLE 1:** Comparison of dosimetric parameters between VMAT and RAD plans.

Structure name	DVH parameter	Clinical goals	VMAT median [Range]	RAD median [Range]	Paired difference median [Range] (VMAT‐RAD)	WSR test p
PTV_TOTAL_	HI (D2–D98)	−	13.0 [12.4, 16.1]	12.8 [9.8, 19.5]	1.0 [−3.4, 3.3]	0.10
PTVp_L	V105%	≤ 10.0%	1.6 [0.4, 7.1]	0.4 [0.0, 11.2]	3.2 [−6.9, 9.8]	0.04
PTVp_R	V105%	≤ 10.0%	2.0 [0.3, 11.1]	0.1 [0.0, 14.2]	3.8 [−11.0, 12.3]	0.02
Esophagus	D0.03 cm^3^	< 30.00 Gy	30.4 [28.3, 32.0]	25.2 [22.2, 28.4]	5.4 [2.5, 8.5]	0.002
Esophagus	Dmean	< 10.00 Gy	10.2 [9.8, 11.8]	9.3 [8.3, 10.2]	1.4 [0.3, 2.5]	0.002
Heart	D5%	< 25.00 Gy	6.1 [3.8, 28.6]	5.7 [3.9, 30.2]	−0.4 [−1.7, 1.6]	0.02
Heart	Dmean	≤ 5.00 Gy	3.1 [1.9, 17.0]	3.2 [2.1, 16.8]	−0.2 [−0.8, 1.4]	0.04
Heart	V15Gy	≤ 30.0%	0.4 [0.0, 11.2]	0.5 [0.0, 10.9]	0.1 [−0.8, 1.7]	0.35
LAD	D0.03 cm^3^	< 7.00 Gy	9.1 [4.7, 27.8]	9.5 [5.3, 26.9]	0.4 [−0.6, 7.7]	0.05
Lungs	V10Gy	≤ 50.0%	35.6 [28.1, 40.2]	32.9 [24.0, 47.4]	2.1 [−11.8, 7.0]	0.10
Lungs	V20Gy	≤ 30.0%	20.8 [14.9, 22.7]	19.4 [13.3, 29.7]	0.3 [−7.8, 4.5]	0.20
Lungs	V5Gy	≤ 65.0%	55.6 [45.7, 63.3]	47.0 [36.0, 58.9]	9.7 [0.9, 16.3]	<0.001
Spinal cord	D0.03 cm^3^	< 45.00 Gy	27.1 [26.6, 38.5]	37.2 [20.4, 39.6]	−6.5 [−10.5, 6.4]	0.02
Body	D0.03 cm^3^	≤ 107.0%	110 [109.2, 112.7]	109.0 [105.8, 112.0]	0.92 [−1.35, 3.39]	0.04
Body	V100%	cc	1683 [884, 3112]	1257 [955, 3590]	236 [−477, 895]	0.20

Cardiac sparing showed minimal clinically significant differences. Although statistically significant reductions in Heart D5% and D_mean_ were noted with VMAT, the median differences were within 0.5 Gy. Heart V15Gy remained nearly identical between the two techniques (*p* = 0.35).

Similar trends were seen for target dose: differences between RAD and VMAT plans were small, with RAD slightly better in PTVp dosimetry and VMAT slightly better in PTVn. D2% of PTV_TOTAL_ is marginally better with RAD compared to VMAT, although no difference was observed for HI (D2‐D98). Detailed dosimetric data, including median values, ranges, and *p* values for all parameters, are presented in Table 1.

Beyond dosimetry, RAD offered substantial efficiency gains. Planning time was markedly reduced with RAD compared to VMAT (median 16.0 vs 39.0 min). Delivery efficiency was also significantly enhanced: RAD plans required approximately 30% fewer Monitor Units (2268 ± 335 for VMAT vs. 1654 ± 229 for RAD); and an averaged 62% shorter total delivery time for the three cases tested on the machine (6.3, 5.5, 5.6 min for VMAT vs. 2.0, 2.5, 2.1 for RAD). The substantially reduced delivery time could translate into fewer breath‐holds in the setting of deep inspiration breath hold (DIBH) treatment and overall improved patient comfort during treatment.

All 10 RAD deliveries achieved 3 %/3 mm gamma pass rates of 99.2%–99.9% (mean 99.6 %) and 2 %/2 mm pass rates of 95.0%–99.4 % (mean 98.2%), comparable to previously reported VMAT breast portal dosimetry passing rates for hybrid breast plans (Koo et al., 2021). Trajectory log file analysis showed minimal mechanical difference between expected and actual motion: ranges aggregated over all logs are −0.179° to +0.147° for gantry, −0.117° to +0.114° for collimator rotations, and −0.080 to +0.087 mm for MLC motions, with 99% differences below 0.1 degree for gantry, 0.1 degree for collimator rotation, and 0.06 mm for MLC.

## 4 DISCUSSION

This study demonstrates that RAD, using only a single arc with six STAMPs, offers significant dosimetric and efficiency advantages over conventional multi‐arc VMAT (VMAT) for the complex challenge of treating SBBC. While maintaining comparable target coverage (V95%) and improving dose homogeneity (reduced V105% in PTVp), RAD achieved notable reductions in critical OAR doses, particularly for the lungs and esophagus. The improved homogeneity can be attributed in part to RAD's integrated auto skin flash tool, which automatically applies virtual bolus with density that varies relative to beam‐skin incidence angle to promote more superficial dose being delivery by tangential beamlets. Unlike manual skin flash techniques for VMAT which often require time‐consuming steps to add‐optimize‐remove virtual bolus, and plan re‐normalization to ensure adequate coverage at the skin surface, this approach removed manual steps, and resulted in more balanced dose distribution throughout the target volume while ensuring robust coverage during potential patient motion or setup uncertainties.^17^ This integration of advanced skin flash technology with simultaneous optimization of arc and STAMP components represents a key technical advantage of the RAD approach.

The most striking dosimetric improvements were observed in lung and esophageal sparing. RAD yielded a statistically significant reduction in the median lung V5Gy by nearly 10% (*p* < 0.001) compared to VMAT. Minimizing the low‐dose bath to the lungs, particularly the V5Gy, is clinically crucial as it may help reduce risk of pneumonitisi^22^. Furthermore, RAD significantly lowered the high‐dose exposure to the esophagus, evidenced by a median reduction in D0.03 cm^3^ of 5.4 Gy (*p* = 0.002). This reduction in the near‐maximum esophageal dose could translate into a lower incidence or severity of acute esophagitis, improving patient tolerance to treatment.^23^ Similarly, improvement in dose homogeneity for breast has been found to reduce acute dermatitis and improve cosmetic outcome.^24^

### 4.1 RAD: Optimization efficiency

The reduction in planning time with RAD (median 16 vs. 39 min for VMAT) represents a significant practical advantage in resource‐limited clinical environments, potentially reducing planner workload and expediting treatment starts. More importantly, the dramatic reduction in estimated total delivery time (∼2  vs. ∼ 5 min, a 62% decrease) and monitor units (avg 1654 vs. 2268 MU, a 30% decrease) has direct patient benefits. Shorter treatment times make breath‐hold treatment more tolerable, enhance patient comfort, reduce the likelihood of intra‐fraction motion affecting dose accuracy^11^ and also increase the capacity for patient throughput. This efficiency gain aligns with findings from other studies investigating hybrid or advanced VMAT techniques.^8^, ^9^, ^10^

### 4.2 Comparison with previous studies and other techniques

Our findings align with preliminary reports on RAD presented recently^16^, ^18^, ^19^ and support the general conclusions from systematic reviews that hybrid techniques can offer dosimetric benefits in breast RT^8^ Specifically, our results echo findings by Lin et al.^10^ who found H‐VMAT (combining tangential IMRT with VMAT arcs) provided better dose homogeneity and OAR sparing (heart, contralateral lung/breast) compared to pure VMAT or F‐IMRT for left‐sided breast cancer. Similarly, Zhang et al.^11^ found H‐VMAT offered a good balance between target coverage, OAR dose, and robustness for post‐mastectomy radiotherapy including internal mammary nodes, although their H‐VMAT involved tangential fields rather than STAMPs.

Comparing RAD to helical tomotherapy (HT) for SBBC, based on the limited data available,^25^, ^26^ suggests potential advantages for RAD. While both techniques achieve excellent target coverage and good homogeneity, RAD appears to offer superior OAR sparing, particularly for the heart (median MHD 3.2  vs. ∼10.7 Gy reported for HT^25^) and low‐dose lung volume (median V5Gy 47.0% vs. ∼55.5% for HT^25^). Furthermore, the delivery efficiency of RAD (median ∼2.25 min) is substantially better than the reported beam‐on times for HT (average ∼11.8 min), representing a significant clinical workflow advantage. The specific advantage of RAD appears to be the combination of dosimetric quality *and* workflow efficiency stemming from its integrated, simultaneous optimization strategy, as well as more generalizable class solutions for each treatment site.

### 4.3 Limitations

This study has several limitations. First, its retrospective nature and small sample size (*N* = 10) may limit generalizability; results require validation in larger, prospective cohorts. Second, this is purely a dosimetric planning study; clinical outcomes like toxicity and tumor control were not assessed, although the dosimetric improvements strongly suggest potential clinical benefits. Finally, the optimal number and arrangement of STAMPs for SBBC also warrant further investigation.

Despite these limitations, our results strongly support RAD as a promising technique for SBBC. Future research should include prospective clinical trials evaluating toxicity and efficacy. Multi‐institutional studies are needed to confirm robustness. Further technical investigations could explore optimizing RAD parameters (number/angle of STAMPs, use of dynamic collimator rotation) specifically for SBBC and compare RAD directly against other advanced platforms like helical tomotherapy or alternative hybrid strategies within the same patient datasets.

## 5 CONCLUSION

RAD demonstrates significant advantages over conventional VMAT for SBBC treatment planning. By integrating static and dynamic beams through simultaneous optimization of the arc and STAMPs, RAD achieves clinically meaningful reductions in low‐dose lung irradiation (V5Gy) and high‐dose esophageal exposure (D0.03 cm^3^) while maintaining excellent target coverage and improving homogeneity. Coupled with substantial improvements in planning and delivery efficiency, RAD emerges as a highly favorable technology, potentially reducing treatment‐related toxicities and improving clinical workflow. Further clinical validation is warranted, but RAD represents a compelling advancement for managing these complex cases.

## AUTHOR CONTRIBUTIONS

TL, LC, SB conceived the study, TL, LR, RC, PA performed treatment planning, TL and SB performed data analysis, TL wrote the manuscript, YG, YY, BKKT, AM, HK, FR and PK reviewed and edited the manuscript.

## CONFLICT OF INTEREST STATEMENT

Ethics approval and consent to participate: The use of the deidentified image data was approved by the ethics committee at Humanitas Cancer Center and Research Hospital, where consent to participate was obtained for all subjects.

Human Ethics declaration: not applicable.

L. Cozzi, L. Rosa, R. Clark, P. Agarwal, A. Magliari, and H. Kalra are employees of Varian Medical Systems. Other authors declare no conflicts of interest.

## Data Availability

The datasets generated and/or analyzed during the current study are not publicly available due to patient privacy concerns but are available from the corresponding author on reasonable request.
